# Structural and biophysical analysis of interactions between cod and human uracil-DNA *N*-glycosylase (UNG) and UNG inhibitor (Ugi)

**DOI:** 10.1107/S1399004714011699

**Published:** 2014-07-25

**Authors:** Netsanet Gizaw Assefa, Laila Niiranen, Kenneth A. Johnson, Hanna-Kirsti Schrøder Leiros, Arne Oskar Smalås, Nils Peder Willassen, Elin Moe

**Affiliations:** a Department of Chemistry/Norstruct, UiT The Arctic University of Norway, 9037 Tromsø, Norway; b Department of Biochemistry, University of Turku, FIN-20014 Turku, Finland; c Instituto de Tecnologia Quimica e Biologica (ITQB), Universidade Nova de Lisboa, Avenida da Republica (EAN), 2780-157 Oeiras, Portugal

**Keywords:** cold adaptation, uracil-DNA *N*-glycosylase, Atlantic cod, protein–inhibitor complex, protein–protein interactions, isothermal titration calorimetry, binding affinity

## Abstract

Uracil-DNA *N*-glycosylase from Atlantic cod (cUNG) shows cold-adapted features such as high catalytic efficiency, a low temperature optimum for activity and reduced thermal stability compared with its mesophilic homologue human UNG (hUNG). In order to understand the role of the enzyme–substrate interaction related to the cold-adapted properties, the structure of cUNG in complex with a bacteriophage encoded natural UNG inhibitor (Ugi) has been determined. The interaction has also been analyzed by isothermal titration calorimetry (ITC). The crystal structure of cUNG–Ugi was determined to a resolution of 1.9 Å with eight complexes in the asymmetric unit related through noncrystallographic symmetry. A comparison of the cUNG–Ugi complex with previously determined structures of UNG–Ugi shows that they are very similar, and confirmed the nucleotide-mimicking properties of Ugi. Biophysically, the interaction between cUNG and Ugi is very strong and shows a binding constant (*K*_b_) which is one order of magnitude larger than that for hUNG–Ugi. The binding of both cUNG and hUNG to Ugi was shown to be favoured by both enthalpic and entropic forces; however, the binding of cUNG to Ugi is mainly dominated by enthalpy, while the entropic term is dominant for hUNG. The observed differences in the binding properties may be explained by an overall greater positive electrostatic surface potential in the protein–Ugi interface of cUNG and the slightly more hydrophobic surface of hUNG.

## Abstract 2

A structural and biophysical study of the interactions between cod and human uracil-DNA *N*-glycosylase (UNG) and their inhibitor Ugi is presented. The stronger interaction between cod UNG and Ugi can be explained by a greater positive electrostatic surface potential.

## 1. Introduction   

Uracil may arise in DNA by misincorporation of dUTP instead of dTTP during DNA replication or by cytosine deamination (Krokan *et al.*, 1997 ▶). Uracil damage can have deleterious consequences in an organism if it is left un­repaired. The highly mutagenic U–G lesions can lead to a transversion of G:C to T:A, which can change the genomic sequence. Even though uracil can sufficiently base pair with adenine, its incorporation can also interfere with the DNA interaction of DNA-binding proteins and affect the regulation of gene expression (Visnes *et al.*, 2009 ▶; Kavli *et al.*, 2007 ▶; Zharkov *et al.*, 2010 ▶). Uracil-DNA *N*-glycosylase (UNG) is a DNA-repair enzyme which initiates the removal of uracil in the base-excision repair (BER) pathway by cleaving the *N*-glycosidic bond between the uracil and the deoxyribose backbone. UNGs have been identified from a number of organisms and crystal structures have been determined for UNGs from human (Mol, Arvai, Slupphaug *et al.*, 1995 ▶), herpes simplex virus 1 (Savva & Pearl, 1995 ▶), *Escherichia coli* (Xiao *et al.*, 1999 ▶), Atlantic cod (Leiros *et al.*, 2003 ▶), *Deinococcus radiodurans* (Leiros *et al.*, 2005 ▶) and *Vibrio cholerae* (Raeder *et al.*, 2010 ▶). All six enzymes have similar structures and consist of a classic single-domain α/β-fold with a central four-stranded parallel and twisted β-sheet surrounded by 11 α-helices. The N- and C-termini are on opposite sides of the central β-sheet and the active site is located within a positively charged groove at the C-terminal end of the β-sheet.

One common characteristic of UNGs is their inhibition by Ugi (Zharkov *et al.*, 2010 ▶). Ugi is a small (9.5 kDa), acidic (pI 4.2) and heat-resistant protein encoded by the *Bacillus subtillis* bacteriophages (PBS1 and PBS2), which use uracil instead of thymine as a normal component of their DNA (Wang & Mosbaugh, 1988 ▶; Cone *et al.*, 1980 ▶). Ugi inhibits UNGs from a wide range of organisms and can form a very stable complex which cannot be disrupted under normal physiological conditions (Acharya *et al.*, 2002 ▶; Bennett *et al.*, 1993 ▶).

**Figure 1:**
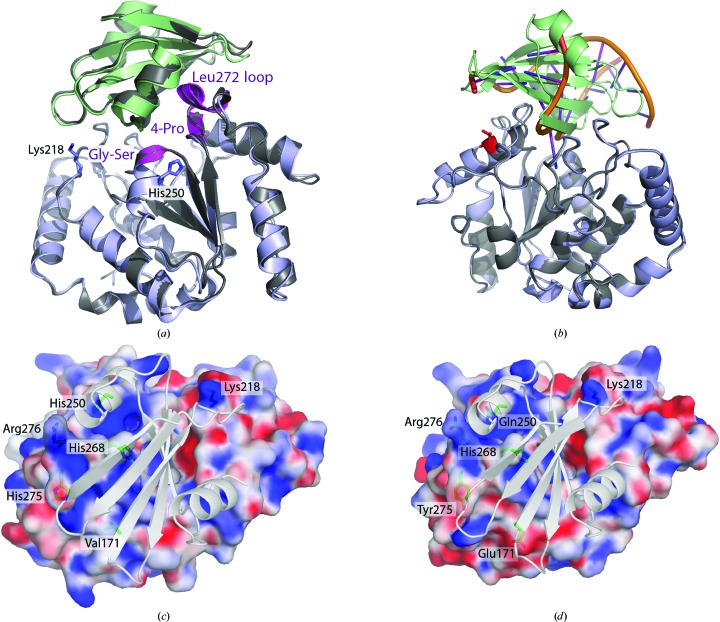
(*a*) The cUNG–Ugi complex (cUNG in blue, Ugi in green) superimposed on the hUNG–Ugi complex (PDB entry 1emh; grey) with some loops labelled. There are no large conformational shifts in cUNG–Ugi compared with hUNG–Ugi, except for in some flexible loop areas. (*b*) Ribbon representation of the cUNG–Ugi complex (cUNG in light blue, Ugi in green) superimposed on the hUNG (in grey) complex structure with uncleaved substrate (uracil) containing DNA (PDB entry 1emh). (*c*, *d*) Calculated electrostatic surface potential of (*c*) cUNG (chains EF) and (*d*) hUNG in complex with Ugi (grey). Selected residues are displayed as stick models and labelled. UNG surfaces are gradient coloured according to the electrostatic potential at 25°C (−12*kT*/e to +12*kT*/e) from negative (red) to positive (blue) potential. The difference in the surface potential of cUNG can be seen as small changes spread over the contact surface area, in particular in the groove accommodating the Ugi β-sheet that mimics the phosphate backbone of DNA.

The crystal structure of UNG in complex with Ugi has been determined for UNGs from human (Mol, Arvai, Sanderson *et al.*, 1995 ▶), herpes simplex virus 1 (Savva & Pearl, 1995 ▶), *E. coli* (Sai­krishnan *et al.*, 2002 ▶; Ravishankar *et al.*, 1998 ▶; Putnam *et al.*, 1999 ▶), Epstein–Barr virus (Géoui *et al.*, 2007 ▶) and *Mycobacterium tuberculosis* (Kaushal *et al.*, 2008 ▶). These complex structures revealed that the UNG–Ugi interaction closely resembles the interaction of UNG with DNA (Mol, Arvai, Sanderson *et al.*, 1995 ▶; Putnam *et al.*, 1999 ▶; Savva & Pearl, 1995 ▶); thus, investigation of the UNG–Ugi interaction appears to be useful for understanding the nature of the UNG–DNA interaction. Ugi binds to the same conserved regions of UNG which normally interact with DNA: the 4-Pro loop (165-PPPPS-169), the Gly-Ser loop (246-GS-247) and the minor-groove intercalation loop (Leu272 loop; 268-HPSPLSVY-275) (hUNG numbering; Fig. 1 ▶ *a*).

Ugi consists of a five-stranded antiparallel β-sheet and two α-helices. It blocks access to the active site of UNG by inserting its β1 strand into the conserved DNA-binding groove of the enzyme without contacting the uracil-specificity pocket (Mol, Arvai, Sanderson *et al.*, 1995 ▶; Putnam *et al.*, 1999 ▶). The interface between hUNG and Ugi involves 12 enzyme side chains and 14 inhibitor side chains, which explains the profound stability of the complex, which cannot be disrupted under physiological conditions (Bennett *et al.*, 1993 ▶; Mol, Arvai, Sanderson *et al.*, 1995 ▶). Even though the mechanism of Ugi binding by UNGs from different organisms is similar, there are differences in the binding affinity (Acharya *et al.*, 2003 ▶; Kaushal *et al.*, 2008 ▶; Géoui *et al.*, 2007 ▶). This has been suggested to be caused by differences in the number of hydrogen bonds, hydrophobic interactions and other electrostatic interactions.

The catalytic domain of UNG from Atlantic cod has previously been identified, recombinantly produced in *E. coli*, purified and characterized, and its crystal structure has been determined (Lanes *et al.*, 2002 ▶; Leiros *et al.*, 2003 ▶; Moe *et al.*, 2004 ▶). Biochemical characterization experiments and molecular-dynamics simulation data have shown that cUNG has characteristic cold-adapted features such as a low temperature optimum for activity, high catalytic efficiency, reduced temperature stability and greater structural elasticity compared with recombinant human UNG (Lanes *et al.*, 2002 ▶; Olufsen *et al.*, 2005 ▶). Indeed, differential scanning calorimetry (DSC) studies of cUNG and hUNG have shown that cUNG is less thermostabile than hUNG (Assefa *et al.*, 2012 ▶). In addition, the crystal structure has shown that cUNG has a more positive electrostatic surface potential in the substrate-binding site (Leiros *et al.*, 2003 ▶; Moe *et al.*, 2004 ▶), which may explain the increased substrate affinity documented for the enzyme by biochemical studies (Lanes *et al.*, 2000 ▶, 2002 ▶). The presence of the nonpolar Val171 on the surface of cUNG instead of a negatively charged Glu as in hUNG has been identified as crucial in this respect (Moe *et al.*, 2004 ▶).

Here, we present the 1.9 Å resolution crystal structure of cUNG in complex with Ugi and the experimental binding analysis of cUNG and hUNG with Ugi. We have performed both comparative structural and isothermal titration calorimetry (ITC) analyses in order to address the question of how the enzyme–substrate interaction may have been optimized for the cold environment of cUNG. Our results show that Ugi is bound more tightly by cUNG than by hUNG at 25°C. Additionally, the contribution of enthalpy to the binding free energy is larger in the cUNG–Ugi association than in the hUNG–Ugi interaction, where the entropic term is dominant. The crystal structure of the cUNG–Ugi complex allows us to explain the greater binding enthalpy by the optimized positive electrostatic surface potential both in and adjacent to the Ugi-binding site of cUNG. Also, the larger entropic contribution in hUNG–Ugi binding is explained by the more hydrophobic hUNG surface.

## 2. Materials and methods   

### 2.1. Expression and purification of cUNG, hUNG and Ugi   

Expression and purification of the UNGs were performed as described previously (Assefa *et al.*, 2012 ▶). The Ugi recombinant plasmid pRSETb, which was kindly provided by Dr Thibault Géoui (EMBL, Grenoble), was used to transform *E. coli* BL21 (DE3) pLysS cells. The purification and expression procedures were modified from Bennett & Mosbaugh (1992 ▶). In brief, the Ugi protein was expressed at 37°C and the cells were harvested after 2–3 h induction with 1 m*M* IPTG. The cells were suspended in 25 m*M* Tris–HCl pH 7.5, 10 m*M* NaCl, 1 m*M* EDTA, 1% glycerol and subjected to ultrasonication. The supernatant was collected by centrifuging the cell lysate for 15 min at 48 000*g*. The supernatant was then heated to 95°C for 15 min and centrifuged for 1 h at 48 000*g*. The resulting supernatant was applied onto a Q-Sepharose column and then onto a Superdex 75 column. The Ugi protein solution was concentrated and stored at −20°C.

### 2.2. Preparation of the cUNG–Ugi complex   

The cUNG–Ugi complex was prepared by mixing cUNG and Ugi in a 1:2 ratio (excess Ugi) and diluting the solution to approximately 3 mg ml^−1^ in buffer consisting of 25 m*M* Tris–HCl, 10 m*M* NaCl, 1 m*M* EDTA, 1% glycerol pH 7.5. The proteins were allowed to bind for 10 min at room temperature followed by 20 min at 4°C. Subsequently, the solution was applied onto a 1 ml HiTrap Q column (GE Healthcare) to separate the cUNG–Ugi complex from unbound Ugi and cUNG. The purified complex was concentrated and stored at 4°C.

### 2.3. Crystallization, data collection, structure determination and analysis   

The cUNG–Ugi complex was crystallized using the hanging-drop method at 4°C with 7.5 mg ml^−1^ protein complex in 25 m*M* Tris–HCl pH 7.5, 10 m*M* NaCl, 1 m*M* EDTA. Drops were made by mixing 1 µl protein with 0.2 µl 0.1 *M* NaBr and 0.8 µl reservoir solution consisting of 0.1 *M* Tris–HCl pH 7.4, 270 m*M* Li_2_SO_4_, 4% PEG 550 MME, 17% PEG 4000. A crystal of about 200 × 200 × 50 µm in size was transferred to a cryoprotectant solution made up of 17% PEG 4000, 10% glycerol and the other reservoir additives at their original concentrations and was then flash-cooled in liquid nitrogen.

Diffraction data were collected on beamline ID-29 at the European Synchrotron Radiation Facility (ESRF), Grenoble, France (de Sanctis *et al.* 2012 ▶) using an ADSC Quantum 315r detector. The crystal belonged to space group *P*2_1_, with unit-cell parameters *a* = 98.21, *b* = 86.92, *c* = 175.37 Å, β = 90.35°, with pseudosymmetric translation (*x* + 0.17, *y* + 1/2, *z* + 0.17) and twinning (−*h*, −*k*, *l*; twin fraction 0.235). There are eight complexes (16 molecules) in the asymmetric unit, giving a solvent content of 54.8% and a Matthews coefficient of 2.7 Å^3^ Da^−1^.

The data were indexed, integrated, scaled and converted to structure factors using the *XDS* program package (Kabsch, 1993 ▶). Two related but not congruent lattices were observed in the data, resulting in many overlapped reflections. It was necessary to decrease the value of the WFAC1 parameter in *XDS* in order to increase the number of misfits rejected before scaling and merging of the data. The structure was solved by molecular replacement using *MOLREP* (Vagin & Teplyakov, 2010 ▶) in *CCP*4 without the PST vector information. The search model was a model of the cUNG–Ugi complex made by superimposing cUNG (PDB entry 1okb; Leiros *et al.*, 2003 ▶) on the structure of the hUNG–Ugi complex (PDB entry 1ugh; Mol, Arvai, Sanderson *et al.*, 1995 ▶). The structure was refined in *REFMAC*5 (Murshudov *et al.*, 2011 ▶) using amplitude-based twin refinement (and no TLS refinement) interspersed with rounds of manual model building in *Coot* (Emsley *et al.*, 2010 ▶). Automatically generated NCS restraints were used in the first ten refinement cycles only, whereas twin refinement was used in all steps. The final model had *R*_work_ and *R*_free_ values of 23.7 and 28.3%, respectively, with acceptable geometry, and was validated using *MolProbity* (Chen *et al.*, 2010 ▶). Details of the data-collection and refinement statistics are given in Table 1 ▶.

The UNG–Ugi interfaces of cUNG–Ugi, hUNG–Ugi (PDB entry 1ugh) and herpes simplex virus 1 UNG–Ugi (PDB entry 1udi; Savva & Pearl, 1995 ▶) were analyzed with the *Protein Interfaces, Surfaces and Assemblies* service (*PISA*; Krissinel & Henrick, 2007 ▶). In addition, the *WHAT IF* web interface (Vriend, 1990 ▶) was used to identify interface electrostatic interactions with interatomic distances of <6 Å. The figures were generated using *PyMOL* (http://www.pymol.org/) and the electrostatic surface potential was calculated using the *APBS* plugin in *PyMOL* (Baker *et al.*, 2001 ▶). The accessible surface area (ASA) was calculated with *Surface Racer* 5.0 (Tsodikov *et al.*, 2002 ▶) with a probe radius of 1.40 Å. cUNG (chain *E*) and hUNG (chain *E*) from the UNG–Ugi complexes were used.

### 2.4. Binding studies by ITC   

ITC measurements were performed at 25°C (standard ITC conditions) using a Nano-ITC III calorimeter from Calorimetry Sciences Corporation (CSC; Utah, USA) with a cell volume of 997 µl. The UNG concentration in the cell was 20–50 µ*M* and the Ugi concentration in the syringe was 267–667 µ*M*. The Ugi solution was injected into the UNG solution as 5 µl aliquots with 300 s intervals between injections and stirring at 150 rev min^−1^. All experiments were performed in 20 m*M* sodium phosphate pH 7.5, 200 m*M* sodium chloride. Buffer exchange with this buffer was performed either by gel filtration or by dialysis using Pierce Slide-A-Lyzer dialysis cassettes from Thermo Fisher Scientific Inc. (Rockford, USA) with a 3 kDa cutoff followed by filtration using a 0.2 µm syringe filter (Millipore, Billerica, USA). The concentration of the protein samples was determined with a NanoDrop 2000c spectrophotometer (NanoDrop Technologies, Wilmington, Delaware, USA) using absorbance at 280 nm and a calculated extinction coefficient for each protein.

The heat of dilution of Ugi in the solvent was measured in a separate experiment. Raw data were integrated, corrected for nonspecific heats, normalized for concentration and analyzed using the standard single-binding-site model in the *Nano­Analyze* program supplied by CSC. The heat values per mole of injectant were plotted against the molar ratio of the ligand and the macromolecule in the cell. The values of the binding affinity, *K*_b_, and the binding enthalpy, Δ*H*_b_, were obtained by fitting the raw data to the best-fit curve of the simple one-site binding model. The binding Gibbs free-energy change, Δ*G*_b_, and the binding entropic term, *T*Δ*S*_b_, were then calculated from the relationship Δ*G*_b_ = −*RT*ln*K*_b_ = Δ*H*_b_ − *T*Δ*S*_b_.

The cUNG–Ugi crystal structure has been deposited in the Protein Data Bank as entry 4lyl.

## 3. Results   

Previous results from biochemical and structural analyses have suggested that the increased catalytic efficiency of cUNG compared with hUNG arises from an increased substrate affinity generated by a large positive electrostatic surface potential in the substrate-binding region of cUNG. In order to obtain more detailed information about the enzyme–substrate interactions, we have determined the crystal structure of the cUNG–Ugi complex and measured the Ugi-binding properties of cUNG and hUNG experimentally by ITC.

### 3.1. The crystal structure of cUNG–Ugi was determined to a resolution of 1.9 Å   

The crystal structure of the cUNG–Ugi complex was determined to 1.9 Å resolution with eight cUNG–Ugi complexes in the asymmetric unit, all related by non­crystallographic symmetry. Data processing was complicated by the presence of pseudosymmetrical translation (PST) and a high copy number in the asymmetric unit. Observed overlaps in the data caused the *R*_merge_ values to be very large and the data to be unusable at the start. We used the WFAC1 parameter in *XDS* to solve this problem by reducing the WFAC1 value and thereby increasing the number of misfits. Owing to PST and twinning, the data behaved as if they belonged to space group *P*2, but when processed in this space group they gave only incomplete molecular-replacement solutions which were impossible to refine. After being processed in the correct space group, *P*2_1_, the structure was easily solved by molecular replacement, giving the same solution irrespective of whether the PST vector information was used or not. In refinement, the *R* values fell below 30% in the first ten cycles when twin refinement was used. A twin law was also imposed in all later steps, although its effect was small (an approximately 3% decrease in the *R* values).

The eight cUNG structures are nearly identical except for some flexible surface residues, and the root-mean-square (r.m.s.) deviation on superposition with native cUNG (PDB entry 1okb) is 0.3 Å. Thus, there are no large shifts in the complexed cUNG structure compared with native cUNG. The r.m.s. deviation when superimposing the eight cUNG–Ugi molecules onto each other is on average 0.3 Å, and for hUNG–Ugi is 0.4 Å (PDB entry 1ugh; Fig. 1 ▶ *a*). The largest differences are found in the Ugi molecules, which are very flexible in some loop areas not involved in complex formation (loops 29–35, 48–53 and 74–78), especially in loop 29–35, which has poor electron density and large *B* factors. X-ray data-collection and crystallographic refinement statistics for the cUNG–Ugi structure are given in Table 1 ▶.

### 3.2. Intermolecular electrostatic interactions are more optimized in the cUNG–Ugi complex structure   

The interface between UNG and Ugi was analyzed with *PISA* (Krissinel & Henrick, 2007 ▶). The details of the interactions (the interface area, the energy contribution of each bond and the solvation free-energy gain upon formation of the interface) were analyzed for each of the eight cUNG–Ugi complexes and the average value was used for comparison with the hUNG–Ugi complex (Mol, Arvai, Sanderson *et al.*, 1995 ▶). According to the analysis, the interface area in cUNG–Ugi (average value of 1043 Å^2^) is smaller than that in hUNG–Ugi (1093 Å^2^) (Supplementary Table S1^1^). The interface analysis also shows that the average hydrogen-bond distances are slightly shorter in the cUNG–Ugi structure (2.8 Å compared with 2.9 Å in hUNG) even though most hydrogen bonds are formed between the same residues as in hUNG–Ugi. There also seems to be more alternative hydrogen bonds and side chain-to-main chain hydrogen bonds in cUNG–Ugi, as well as two electrostatic interactions in the cUNG–Ugi interface (Lys218–Asp40 and His250–Glu28) which are not found in hUNG–Ugi (Table 2 ▶ and Supplementary Table S2). In order to ascertain that the detected bond-distance differences were not caused by the use of different refinement programs *(REFMAC* 5.8 *versus X-PLOR* 3.851) during the structure-determination process, the deposited structure factors for PDB entry 1ugh were retrieved from the PDB and the hUNG–Ugi complex structure was re-refined using the same refinement procedure as used for cUNG–Ugi. Although some minor changes were observed compared with the original structure (data not shown), the overall results were nearly identical to the original surface-area and bond-distance calculations.

### 3.3. Ugi mimics the electrostatic and shape complementarity of the DNA substrate   

A superimposition of the hUNG–DNA (PDB entry 1emh; Parikh *et al.*, 2000 ▶) and cUNG–Ugi structures (Fig. 1 ▶ *b*) shows that Ugi binds to the DNA-binding site of UNG and generates the same nonspecific contacts with UNG as made by DNA. As described by Mol, Arvai, Sanderson *et al.* (1995 ▶), Ugi mimics the duplex DNA shape and charge complementarity by making hydrogen bonds to conserved UNG active-site residues and by accommodating the protruding Leu272 in a hydrophobic pocket. In the cUNG–Ugi structure these contacts are further strengthened by shorter distances and additional hydrogen bonds and long-distance electrostatic interactions. For example, both the DNA backbone phosphate-mimicking Ugi residues (Glu20 in the inserted β-sheet and Glu28 in the following α-helix) are bound tightly in cUNG–Ugi (Table 2 ▶).

### 3.4. cUNG binds Ugi more tightly with interactions driven mainly by enthalpic forces   

**Figure 2:**
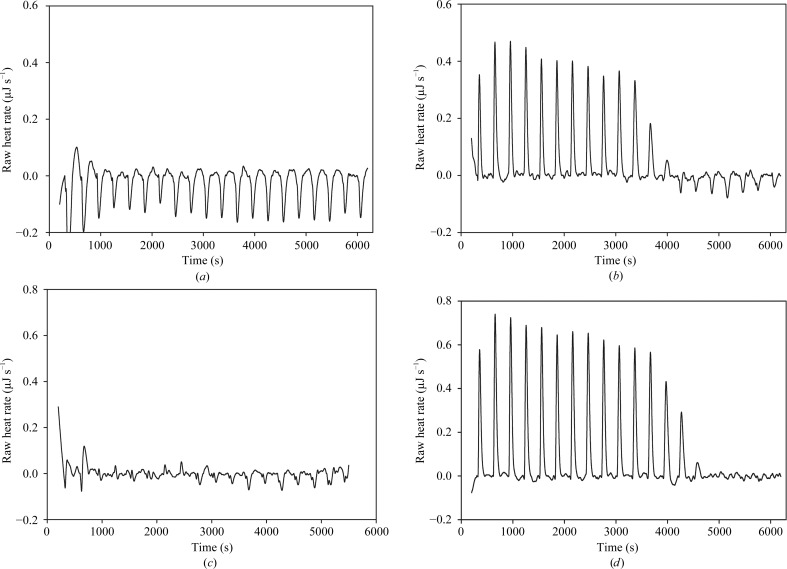
ITC raw heat pulse data for the association of UNG with Ugi at 25°C in 20 m*M* sodium phosphate, 200 m*M* NaCl pH 7.5. The heat was measured upon injecting 20 5 µl aliquots of Ugi into a 997 µl reaction cell containing UNG. 667 µ*M* Ugi was titrated into the buffer in (*a*) and 50 µ*M* hUNG in (*b*); 267 µ*M* Ugi was titrated into the buffer in (*c*) and 20 µ*M* cUNG in (*d*).

**Figure 3:**
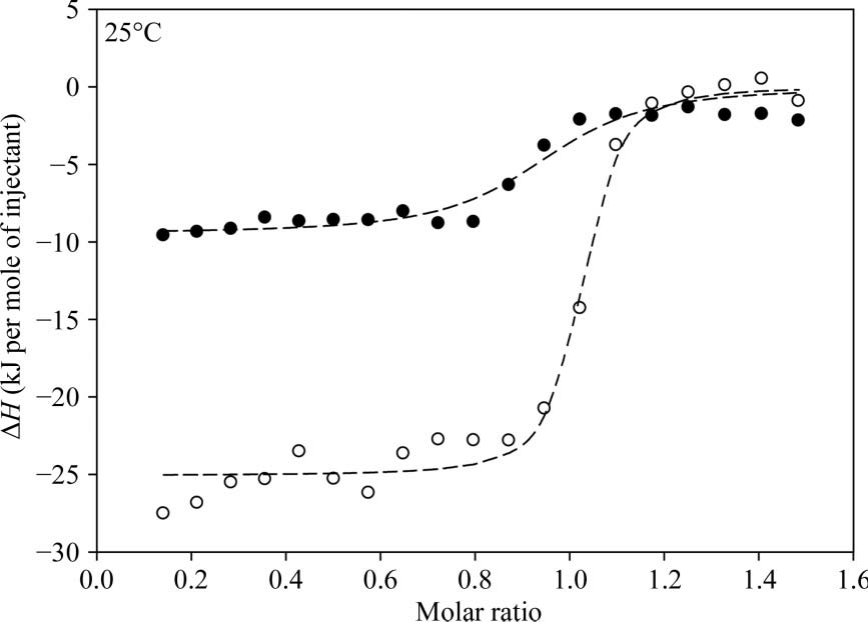
ITC binding isotherm for the association of UNG with Ugi at 25°C in 20 m*M* sodium phosphate, 200 m*M* NaCl pH 7.5: the curves represent the nonlinear least-squares fit (lines) of the affinity (*K*_b_), enthalpy change (Δ*H*_b_) and stoichiometry (*n*) obtained by fitting integrated heat pulse data (scattered points) to a single-site binding model. Key: hUNG, closed circles; cUNG, open circles.

In order to analyze the forces involved in the interaction between cUNG and hUNG and the inhibitor Ugi, their binding properties were measured experimentally by ITC in 20 m*M* sodium phosphate pH 7.5, 200 m*M* NaCl at 25°C. Fig. 2 ▶ shows typical ITC profiles for the association of UNG and Ugi at 25°C. The reaction enthalpy values were corrected for blank runs and for protein concentration and were plotted against the Ugi:UNG molar ratio (Fig. 3 ▶). The binding stoichiometry was approximately 1:1 for both samples. The thermodynamic binding parameters are listed in Table 3 ▶ and show that cUNG binds Ugi more tightly than hUNG, with binding constants (*K*_b_) of 3.25 × 10^7^ and 0.15 × 10^7^ *M* ^−1^, respectively. The magnitude and the negative value of Δ*G*_b_ in both enzymes indicate that the association is strongly favoured from a thermodynamic point of view. The negative values of Δ*H*_b_ and the positive values of *T*Δ*S*_b_ in hUNG and cUNG show that the binding process is both enthalpy-driven and entropy-driven. However, the contribution of Δ*H*_b_ and *T*Δ*S*_b_ to Δ*G*_b_ and thus to *K*_b_ appears to be different in the two enzymes, with the enthalpic contribution dominant in cUNG and the entropic term dominant in hUNG (Table 3 ▶).

## 4. Discussion   

The interaction of UNGs from several species with Ugi has been studied extensively, mainly owing to its potential use as a drug target (Priet *et al.*, 2005 ▶). Binding of Ugi to the conserved DNA-binding groove of UNG by mimicking the shape and electrostatic complementarity of DNA makes it a useful model to study UNG–substrate interactions (Mol, Arvai, Sanderson *et al.*, 1995 ▶). Indeed, in all of the electrostatic interactions in the interface between UNG and Ugi the negatively charged interaction partner is always a Ugi residue and the positively charged interaction partner is always a UNG residue. Whereas most studies have mainly focused on the structural aspects of the UNG–Ugi association, we have here been able to combine structural studies with thermodynamic data of the UNG–Ugi interaction with the intention of extrapolating the findings to UNG–DNA binding. The ease of producing Ugi recombinantly in large amounts and its relative stability under a range of experimental conditions fulfills the requirements for application of ITC.

The cod enzyme cUNG is a cold-adapted enzyme characterized by greater catalytic efficiency and reduced thermal stability compared with its warm-active homologue hUNG. An increased positive electrostatic surface potential in the DNA-binding region of cUNG has been suggested to explain the observed greater catalytic efficiency towards negatively charged DNA substrates (Leiros *et al.*, 2003 ▶; Moe *et al.*, 2004 ▶; Olufsen *et al.*, 2008 ▶). Our ITC results show that the binding constant (*K*_b_) for the association of cUNG with Ugi is one order of magnitude larger than that of hUNG. This observation is in line with the smaller *K*_m_ value (better substrate affinity) previously identified in enzyme-kinetics studies of cUNG compared with hUNG (Lanes *et al.*, 2002 ▶). Cold-adapted enzymes with small *K*_m_ values have a more negative Δ*G*_b_ than those with larger *K*_m_ values (Siddiqui & Cavicchioli, 2006 ▶; Tsuruta & Aizono, 2003 ▶), which is also demonstrated for cUNG–Ugi in this study.

The enthalpy change of the cUNG–Ugi association is larger than that for hUNG–Ugi. One might assume that a large change in Δ*H*_b_ might be caused by significant conformational changes upon binding (Ogasahara *et al.*, 2003 ▶). However, the crystal structure of the cUNG–Ugi complex reveals that the conformation of cUNG is almost identical to the uncomplexed cUNG structure. The observed large enthalpy change must therefore originate from favourable electrostatic or nonpolar interactions between the residues in the interface areas. Indeed, it was observed that the interface area of the cUNG–Ugi complex has a more positive electrostatic potential surface (Figs. 1 ▶ *c* and 1 ▶ *d*) and an increased number of electrostatic interactions compared with that of hUNG–Ugi (PDB entry 1ugh). The larger binding enthalpy of cUNG–Ugi (−25.1 kJ mol^−1^ at 25°C compared with −9.4 kJ mol^−1^ for hUNG–Ugi) clearly indicates that the electrostatic inter­actions in cUNG–Ugi are more stabilizing than those found in the hUNG–Ugi complex. In particular, the Glu171Val, Val185Lys, Gln250His and Tyr275His substitutions in cUNG compared with hUNG result in more favourable interactions with the negatively charged Ugi and the DNA substrate. These findings further support the hypothesis that enhanced intermolecular electrostatic interactions cause the greater binding affinity of cUNG.

As discussed above, the enthalpy-dominated cUNG–Ugi complex formation can be explained by optimized polar contacts and less favourable conformational entropy of the subsequently more stable complex. The buried interface surface area is on average 50 Å^2^ larger in hUNG–Ugi, suggesting that in this case the large entropy term may originate from a more extensive burial of nonpolar groups, leading to a larger desolvation energy gain and thus greater entropy (Supplementary Table S3). Also, the greater conformational entropy of the less stable hUNG–Ugi complex contributes to the entropic domination.

The co-crystal structure of cUNG–Ugi presented in this study supports the previously reported DNA-mimicking properties of Ugi. Determination of the binding affinity by ITC has shown that cUNG binds Ugi more strongly than hUNG. We have learned from previous biochemical studies that cUNG is more catalytically efficient than hUNG towards its U-lesion-containing DNA substrate (Lanes *et al.*, 2000 ▶, 2002 ▶). Based on previous biochemical and structural studies, together with the structural and biophysical analysis from this work, we propose that the high catalytic efficiency of cUNG is mainly caused by its increased substrate-binding affinity through enhanced temperature-adaptive interaction with the DNA substrate, as with the DNA-mimicking inhibitor Ugi.

## Supplementary Material

Supporting Information.. DOI: 10.1107/S1399004714011699/gm5031sup1.pdf

PDB reference: UNG–Ugi complex, 4lyl

## Figures and Tables

**Table 1:** X-ray data-collection and crystallographic refinement statistics for cUNG–Ugi Values in parentheses are for the outer resolution shell.

Data collection	
X-ray source	ID-29, ESRF
Space group	*P*2_1_
Unit-cell parameters (Å, °)	*a* = 98.21, *b* = 86.92, *c* = 175.37, β = 90.35
Resolution (Å)	30–1.94 (2.04–1.94)
Wavelength (Å)	1.0000
No. of unique reflections	199006 (26370)
Multiplicity	2.94 (3.17)
Completeness (%)	91.2 (86.6)
〈*I*〉/〈σ(*I*)〉	10.76 (3.29)
*R*_merge_ † (%)	7.3 (41.3)
Wilson *B* factor (Å^2^)	29.9
Refinement	
*R* factor (all reflections) (%)	23.7
*R*_free_ ‡ (%)	28.3
No. of atoms	21002
No. of water molecules	1483
R.m.s.d., bond lengths (Å)	0.011
R.m.s.d., bond angles (°)	1.669
Average *B* factor (Å^2^)	
All atoms	25.2
Protein	25.0
Water molecules	27.6
Ramachandran plot	
Favoured regions (%)	96.2
Allowed regions (%)	100
PDB code	4lyl

† *R*_merge_ = 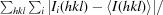 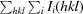, where *I*_*i*_(*hkl*) is the *i*th measurement of reflection *hkl* and 〈*I*(*hkl*)〉 is the weighted mean of all measurements of *hkl*.

‡ 5% of the reflections were used in the *R*_free_ calculations.

**Table 2:** Hydrogen bonds and electrostatic interactions in the *EF* molecule of the cUNG–Ugi and hUNG–Ugi complexes cUNG–Ugi chains *E* and *F* were chosen as a representative of the eight complexes. Hydrogen bonds (<3.5 Å) were analyzed with *PISA*, and the *WHAT IF* web interface was used to identify electrostatic interactions with interatomic distances of <6 Å. Unique bonds are shown in bold.

cUNG–Ugi			hUNG–Ugi		
UNG	Distance (Å)	Ugi	UNG	Distance (Å)	Ugi
Hydrogen bonds					
Gln144 O^∊1^	2.64	Leu23 N	Gln144 O^∊1^	2.97	Leu23 N
Gln144 N^∊2^	2.73	Leu23 O	Gln144 N^∊2^	2.94	Leu23 O
His148 N^∊2^	3.09	Ser21 O^γ^	His148 N^∊2^	2.79	Ser21 O^γ^
Gln152 N^∊2^	2.78	Gln19 O	Gln152 N^∊2^	3.05	Gln19 O
Ser169 N	2.85	Glu20 O^∊1^	Ser169 N	2.98	Glu20 O^∊1^
Ser169 O^γ^	2.56	Glu20 O^∊2^	Ser169 O^γ^	3.06	Glu20 O^∊2^
			**Ala214 O**	**2.84**	**Tyr65 O^η^**
**Ala214** **N**	**2.76**	**Tyr65 O^η^**			
Asn215 N^δ2^	2.76	Asp61 O^δ1^	Asn215 N^δ2^	3.04	Asp61 O^δ1^
Lys218 N^ζ^	2.68	Asp61 ^Oδ2^	Lys218 N^ζ^	2.64	Asp61 O^δ2^
Ser247 N	2.38	Glu28 O^∊2^	Ser247 N	3.08	Glu28 O^∊2^
**Ser247 O^γ^**	**2.76**	**Glu28 O^∊2^**			
Pro271 O	2.57	Gln73 N^∊2^	Pro271 O	2.80	Gln73 N^∊2^
**Leu272 N**	**3.46**	**Met56 S^δ^**			
**Arg276 N^η1^**	**3.27**	**Glu31 O**			
			**Arg276 N^η2^**	**2.98**	**Glu31 O^∊1^**
Average	2.81			2.93	
Electrostatic interactions					
Lys218 N^ζ^	2.68	Asp61 O^δ2^	Lys218 N^ζ^	2.64	Asp61 O^δ2^
**Lys218 N^ζ^**	**5.95**	**Asp40 O^δ2^**			
**His250 N^δ1^**	**5.22**	**Glu28 O^∊2^**			
His268 N^δ1^	5.46	Glu20 O^∊2^	His268 N^δ1^	5.93	Glu20 O^∊2^
			**Arg276 N^η2^**	**2.98**	**Glu31 O^∊1^**
Average	4.83			3.85	

**Table 3:** Thermodynamic binding parameters for the binding of Ugi to cUNG and hUNG at 25°C in 20 m*M* sodium phosphate, 200 m*M* NaCl pH 7.5 The standard deviation of the fit (χ^2^) is given. The standard deviation of the parameters directly measured by the experiment was analysed at the 95% confidence level.

	cUNG	hUNG
Δ*G*_b_ (kJ mol^−1^)	−42.9	−35.2
Δ*H*_b_ (kJ mol^−1^)	−25.1 ± 0.8	−9.4 ± 1.1
*T*Δ*S*_b_ (kJ mol^−1^)	17.8	25.8
*K*_b_ (*M* ^−1^)	(3.25 ± 2.88) × 10^7^	(0.15 ± 0.13) × 10^7^
*K*_d_ (n*M*)	31	685
*n*	0.994 ± 0.013	0.921 ± 0.055
χ^2^	1.69	2.95

## Footnotes

1 Supporting information has been deposited in the IUCr electronic archive (Reference: GM5031).

## References

[bb1] Acharya, N., Kumar, P. & Varshney, U. (2003). *Microbiology*, **149**, 1647–1658. 10.1099/mic.0.26228-0 12855717

[bb2] Acharya, N., Roy, S. & Varshney, U. (2002). *J. Mol. Biol.* **321**, 579–590. 10.1016/s0022-2836(02)00654-x 12206774

[bb3] Assefa, N. G., Niiranen, L., Willassen, N. P., Smalås, A. & Moe, E. (2012). *Comp. Biochem. Physiol. B Biochem. Mol. Biol.* **161**, 60–68. 10.1016/j.cbpb.2011.09.007 21959147

[bb4] Baker, N. A., Sept, D., Joseph, S., Holst, M. J. & McCammon, J. A. (2001). *Proc. Natl Acad. Sci. USA*, **98**, 10037–10041. 10.1073/pnas.181342398 PMC56910 11517324

[bb5] Bennett, S. E. & Mosbaugh, D. W. (1992). *J. Biol. Chem.* **267**, 22512–22521. 1429601

[bb6] Bennett, S. E., Schimerlik, M. I. & Mosbaugh, D. W. (1993). *J. Biol. Chem.* **268**, 26879–26885. 8262921

[bb7] Chen, V. B., Arendall, W. B., Headd, J. J., Keedy, D. A., Immormino, R. M., Kapral, G. J., Murray, L. W., Richardson, J. S. & Richardson, D. C. (2010). *Acta Cryst.* D**66**, 12–21. 10.1107/S0907444909042073 PMC2803126 20057044

[bb8] Cone, R., Bonura, T. & Friedberg, E. C. (1980). *J. Biol. Chem.* **255**, 10354–10358. 6776115

[bb9] Emsley, P., Lohkamp, B., Scott, W. G. & Cowtan, K. (2010). *Acta Cryst.* D**66**, 486–501. 10.1107/S0907444910007493 PMC2852313 20383002

[bb10] Géoui, T., Buisson, M., Tarbouriech, N. & Burmeister, W. P. (2007). *J. Mol. Biol.* **366**, 117–131. 10.1016/j.jmb.2006.11.007 17157317

[bb11] Kabsch, W. (1993). *J. Appl. Cryst.* **26**, 795–800.

[bb12] Kaushal, P. S., Talawar, R. K., Krishna, P. D. V., Varshney, U. & Vijayan, M. (2008). *Acta Cryst.* D**64**, 551–560. 10.1107/S090744490800512X 18453691

[bb13] Kavli, B., Otterlei, M., Slupphaug, G. & Krokan, H. E. (2007). *DNA Repair*, **6**, 505–516. 10.1016/j.dnarep.2006.10.014 17116429

[bb14] Krissinel, E. & Henrick, K. (2007). *J. Mol. Biol.* **372**, 774–797. 10.1016/j.jmb.2007.05.022 17681537

[bb15] Krokan, H. E., Standal, R. & Slupphaug, G. (1997). *Biochem. J.* **325**, 1–16. 10.1042/bj3250001 PMC1218522 9224623

[bb16] Lanes, O., Guddal, P. H., Gjellesvik, D. R. & Willassen, N. P. (2000). *Comp. Biochem. Physiol. B Biochem. Mol. Biol.* **127**, 399–410. 10.1016/s0305-0491(00)00271-6 11126771

[bb17] Lanes, O., Leiros, I., Smalås, A. O. & Willassen, N. P. (2002). *Extremophiles*, **6**, 73–86. 10.1007/s007920100225 11878565

[bb18] Leiros, I., Moe, E., Lanes, O., Smalås, A. O. & Willassen, N. P. (2003). *Acta Cryst.* D**59**, 1357–1365. 10.1107/s0907444903011144 12876336

[bb19] Leiros, I., Moe, E., Smalås, A. O. & McSweeney, S. (2005). *Acta Cryst.* D**61**, 1049–1056. 10.1107/S090744490501382X 16041069

[bb20] Moe, E., Leiros, I., Riise, E. K., Olufsen, M., Lanes, O., Smalås, A. & Willassen, N. P. (2004). *J. Mol. Biol.* **343**, 1221–1230. 10.1016/j.jmb.2004.09.004 15491608

[bb21] Mol, C. D., Arvai, A. S., Sanderson, R. J., Slupphaug, G., Kavli, B., Krokan, H. E., Mosbaugh, D. W. & Tainer, J. A. (1995). *Cell*, **82**, 701–708. 10.1016/0092-8674(95)90467-0 7671300

[bb22] Mol, C. D., Arvai, A. S., Slupphaug, G., Kavli, B., Alseth, I., Krokan, H. E. & Tainer, J. A. (1995). *Cell*, **80**, 869–878. 10.1016/0092-8674(95)90290-2 7697717

[bb23] Murshudov, G. N., Skubák, P., Lebedev, A. A., Pannu, N. S., Steiner, R. A., Nicholls, R. A., Winn, M. D., Long, F. & Vagin, A. A. (2011). *Acta Cryst.* D**67**, 355–367. 10.1107/S0907444911001314 PMC3069751 21460454

[bb24] Ogasahara, K., Ishida, M. & Yutani, K. (2003). *J. Biol. Chem.* **278**, 8922–8928. 10.1074/jbc.m210893200 12643278

[bb25] Olufsen, M., Smalås, A. O. & Brandsdal, B. O. (2008). *J. Mol. Model.* **14**, 201–213. 10.1007/s00894-007-0261-0 18196298

[bb26] Olufsen, M., Smalås, A. O., Moe, E. & Brandsdal, B. O. (2005). *J. Biol. Chem.* **280**, 18042–18048. 10.1074/jbc.M500948200 15749696

[bb43] Parikh, S. S., Walcher, G., Jones, G. D., Slupphaug, G., Krokan, H. E., Blackburn, G. M. & Tainer, J. A. (2000). *Proc. Natl Acad. Sci. USA*, **97**, 5083–5088. 10.1073/pnas.97.10.5083 PMC25785 10805771

[bb27] Priet, S., Gros, N., Navarro, J. M., Boretto, J., Canard, B., Quérat, G. & Sire, J. (2005). *Mol. Cell*, **17**, 479–490. 10.1016/j.molcel.2005.01.016 15721252

[bb28] Putnam, C. D., Shroyer, M. J., Lundquist, A. J., Mol, C. D., Arvai, A. S., Mosbaugh, D. W. & Tainer, J. A. (1999). *J. Mol. Biol.* **287**, 331–346. 10.1006/jmbi.1999.2605 10080896

[bb29] Raeder, I. L. U., Moe, E., Willassen, N. P., Smalås, A. O. & Leiros, I. (2010). *Acta Cryst.* F**66**, 130–136. 10.1107/S1744309109052063 PMC2815677 20124707

[bb30] Ravishankar, R., Bidya Sagar, M., Roy, S., Purnapatre, K., Handa, P., Varshney, U. & Vijayan, M. (1998). *Nucleic Acids Res.* **26**, 4880–4887. 10.1093/nar/26.21.4880 PMC147935 9776748

[bb31] Saikrishnan, K., Bidya Sagar, M., Ravishankar, R., Roy, S., Purnapatre, K., Handa, P., Varshney, U. & Vijayan, M. (2002). *Acta Cryst.* D**58**, 1269–1276. 10.1107/s0907444902009599 12136137

[bb44] Sanctis, D. de *et al.* (2012). *J. Synchrotron Rad.* **19**, 455–461.

[bb32] Savva, R. & Pearl, L. H. (1995). *Nature Struct. Biol.* **2**, 752–757. 10.1038/nsb0995-752 7552746

[bb34] Siddiqui, K. S. & Cavicchioli, R. (2006). *Annu. Rev. Biochem.* **75**, 403–433. 10.1146/annurev.biochem.75.103004.142723 16756497

[bb35] Tsodikov, O. V., Record, M. T. Jr & Sergeev, Y. V. (2002). *J. Comput. Chem.* **23**, 600–609. 10.1002/jcc.10061 11939594

[bb36] Tsuruta, H. & Aizono, Y. (2003). *J. Biochem.* **133**, 225–230. 10.1093/jb/mvg029 12761186

[bb37] Vagin, A. & Teplyakov, A. (2010). *Acta Cryst.* D**66**, 22–25. 10.1107/S0907444909042589 20057045

[bb38] Visnes, T., Doseth, B., Pettersen, H. S., Hagen, L., Sousa, M. M., Akbari, M., Otterlei, M., Kavli, B., Slupphaug, G. & Krokan, H. E. (2009). *Philos. Trans. R. Soc. Lond. B Biol. Sci.* **364**, 563–568. 10.1098/rstb.2008.0186 PMC2660913 19008197

[bb39] Vriend, G. (1990). *J. Mol. Graph.* **8**, 52–56. 10.1016/0263-7855(90)80070-v 2268628

[bb40] Wang, Z. & Mosbaugh, D. W. (1988). *J. BacterioI.* **170**, 1082–1091.

[bb41] Xiao, G., Tordova, M., Jagadeesh, J., Drohat, A. C., Stivers, J. T. & Gilliland, G. L. (1999). *Proteins*, **35**, 13–24. 10090282

[bb42] Zharkov, D. O., Mechetin, G. V. & Nevinsky, G. A. (2010). *Mutat. Res.* **685**, 11–20. 10.1016/j.mrfmmm.2009.10.017 PMC3000906 19909758

