# Supplementary material for: Structural and biophysical analysis of interactions between cod and human uracil-DNA N-glycosylase (UNG) and UNG inhibitor (Ugi)
Source: Acta Crystallogr D Biol Crystallogr. 2014 Jul 25;70(Pt 8):2093–100. doi: 10.1107/S1399004714011699 (PMC4118823; doi:10.1107/S1399004714011699)
Supplement: Supplementary file 1 [file d-70-02093-sup1.pdf]

Acta Crystallographica Section D

Volume 70 (2014)

Supporting information for article:

Structural and biophysical analysis of interactions between cod and human uracil-DNA N-glycosylase (UNG) and UNG inhibitor (Ugi)

Netsanet Gizaw Assefa, Laila Niiranen, Kenneth Johnson, Hanna-Kirsti Schrøder Leiros, Arne Oskar Smalås, Nils Peder Willassen and Elin Moe

Table S1 Protein-protein interface data of hUNG-Ugi and cUNG-Ugi from PISA and WHAT IF calculations.

Protein-Ugi		Interface area (Å^2)	Hydrogen bonds (<3.5Å)	Electrostatic interactions (<6Å)
hUNG-Ugi		1093	12	3
cUNG-Ugi	A+B	1035	14	4
	C+D	1053	12	3
	E+F	1050	14	4
	G+H	1061	14	3
	I+J	1033	13	4
	K+L	1045	12	3
	M+N	1025	13	2
	O+P	1041	10	2
cUNG-Ugi	Average	1043	12.8	3.1

Table S2 Hydrogen bonds and electrostatic interactions in the cUNG-Ugi complex (eight complexes in one asymmetric unit) and hUNG-Ugi. Hydrogen bonds (<3.5 Å) were analyzed with PISA, and the WHAT IF Web Interface was used to identify electrostatic interactions with interatomic distance of <6 Å.

Hydrogen bond distances (Å)

cUNG-Ugi chains									Interacting atoms		cUNG average		hUNG
	AB	CD	EF	GH	IJ	KL	MN	OP	Ugi	UNG	Dist	Stdev	Dist
	2.91	2.86	2.78	3.07			3.30		Gln19 O	Gln152 Nε_2	2.98	0.21	3.05
	3.02	2.94	2.85	2.95	2.69	2.74	2.81	2.74	Glu20 Oε_1	Ser169 N	2.84	0.12	2.98
	2.72	2.47	2.56	2.64	2.75	2.51	2.64	2.61	Glu20 Oε_2	Ser169 Oγ	2.61	0.10	3.06
	3.09	2.66	3.09	3.04	3.20	3.01	3.14	2.97	Ser 21 Oγ	His148 Nε_2	3.03	0.16	2.79
	2.82	2.66	2.64	2.75	2.57	2.74	2.85	2.59	Leu23 N	Gln144 Oε_1	2.70	0.10	2.97
	3.04	2.80	2.73	3.11	3.09	3.09	2.96	3.06	Leu23 O	Gln144 Nε_2	2.99	0.14	2.94
	2.96	2.65	2.38	3.05	2.97	2.88	2.74	2.85	Glu28 Oε_2	Ser247 N	2.81	0.22	3.08
			2.76						Glu28 Oε_2	Ser247 Oγ	2.76		
			3.27						Glu31 O	Arg276 Nη_1	3.27		
					2.34	2.97			Glu31 Oε_1	Arg276 Nη_2	2.66	0.45	2.98
	3.38		3.46	3.38	3.42	3.45	3.35		Met56 Sδ	Leu272 N	3.41	0.04	
	2.96	3.29	2.76	3.04	2.63	2.76	2.84	2.83	Asp61 Oδ_1	Asn215 Nδ_2	2.89	0.21	3.04
	3.24							3.10	Asp61 Oδ_2	Asn215 Nδ_2	3.17	0.10	
	2.70	2.82	2.68	3.02	2.67	2.98	2.79	3.10	Asp61 Oδ_2	Lys218 Nζ	2.85	0.17	2.64
	3.47			2.43					Ala62 O	Lys218 Nζ	2.95	0.74	
	2.83	3.46	2.76	3.38	3.27		3.24		Tyr65 Oη	Ala214 N	3.16	0.29	
		2.55		2.76	2.68	2.77	2.71	2.50	Tyr65 Oη	Ala214 O	2.66	0.11	2.84
	2.82	2.80	2.57	2.67	2.78	2.58	2.76	3.04	Gln73 Nε_2	Pro271 O	2.75	0.15	2.80
Aver.	3.00	2.83	2.81	2.95	2.85	2.87	2.93	2.85			2.92	0.07	2.93

Electrostatic interaction distances (Å)

cUNG-Ugi chains									Interacting atoms		cUNG average		hUNG
	AB	CD	EF	GH	IJ	KL	MN	OP	Ugi	cUNG	Dist	Stdev	Dist
	5.53	5.52	5.46	5.53	5.42	5.35	5.58	5.54	Glu20 Oε_2	His268 Nδ_1	5.49	0.08	5.93
			5.22						Glu28 Oε_2	His250 Nδ_1	5.22		
					2.34				Glu31 Oε_1	Arg276 Nη_1	2.34		
	4.36								Glu31 Oε_1	Arg276 Nη_2	4.36		2.98
				4.41		2.97			Glu31 Oε_2	Arg276 Nη_2	3.69	1.02	
		5.65							Asp40 Oδ_2	Lys251 Nζ	5.65		
	5.95		5.95		5.85				Asp40 Oδ_2	Lys218 Nζ	5.92	0.06	
	2.70	2.82	2.68	3.02	2.67	2.98	2.79	3.10	Asp61 Oδ_2	Lys218 Nζ	2.85	0.17	2.64
Aver.	4.64	4.66	4.83	4.32	4.07	3.77	4.19	4.32			4.44	0.33	3.85

Table S3 Calculated accessible surface area (ASA) with Surface Racer 5.0 for cUNG (chain E) and hUNG (chain E) from the UNG-UGI complexes.

Protein	cUNG	hUNG	Difference
Chain	E	E	(cUNG – hUNG)
PDB	4LYL	1UGH	
TOTAL ASA	10376	10497	-122
Polar ASA	4838	4831	7
Non-polar ASA	5537	5666	-129
Polar back bone ASA	1241	1294	-53
Non-polar back bone	690	807	-117
Polar side chain ASA	3597	3537	61
Non-polar side chain ASA	4847	4859	-12
+charge ASA	1574	1548	26
-charge ASA	849	690	159
